# Supersaturated Lipid-Based Formulations to Enhance the Oral Bioavailability of Venetoclax

**DOI:** 10.3390/pharmaceutics12060564

**Published:** 2020-06-18

**Authors:** Niklas J. Koehl, Laura J. Henze, Martin Kuentz, René Holm, Brendan T. Griffin

**Affiliations:** 1 School of Pharmacy, University College Cork, College Road, T12 YN60 Cork, Ireland; Niklas.Koehl@ucc.ie (N.J.K.); Laura.Henze@ucc.ie (L.J.H.); 2 Institute of Pharma Technology, University of Applied Sciences and Arts Northwestern Switzerland, Hofackerstrasse 30, 4132 Muttenz, Switzerland; Martin.Kuentz@fhnw.ch; 3 Drug Product Development, Janssen Research and Development, Johnson & Johnson, Turnhoutseweg 30, 2340 Beerse, Belgium; rholm@ITS.JNJ.com; 4 Department of Science and Environment, Roskilde University, 4000 Roskilde, Denmark

**Keywords:** lipid-based formulation, venetoclax, Super-SNEDDS, supersaturation, amorphous solubility, landrace pigs, supersaturated lipid-based formulations, lipid suspensions, self-emulsifying drug delivery system, SEDDS

## Abstract

Increasing numbers of beyond Rule-of-Five drugs are emerging from discovery pipelines, generating a need for bio-enabling formulation approaches, such as lipid-based formulations (LBF), to ensure maximal in vivo exposure. However, many drug candidates display insufficient lipid solubility, leading to dose-loading limitations in LBFs. The aim of this study was to explore the potential of supersaturated LBFs (sLBF) for the beyond Rule-of-Five drug venetoclax. Temperature-induced sLBFs of venetoclax were obtained in olive oil, Captex^®^ 1000, Peceol^®^ and Capmul MCM^®^, respectively. A Peceol^®^-based sLBF displayed the highest drug loading and was therefore evaluated further. In vitro lipolysis demonstrated that the Peceol^®^-based sLBF was able to generate higher venetoclax concentrations in the aqueous phase compared to a Peceol^®^-based suspension and an aqueous suspension. A subsequent bioavailability study in pigs demonstrated for sLBF a 3.8-fold and 2.1-fold higher bioavailability compared to the drug powder and Peceol^®^-based suspension, respectively. In conclusion, sLBF is a promising bio-enabling formulation approach to enhance in vivo exposure of beyond Rule-of-Five drugs, such as venetoclax. The in vitro lipolysis results correctly predicted a higher exposure of the sLBF in vivo. The findings of this study are of particular relevance to pre-clinical drug development, where maximum exposure is required.

## 1. Introduction

In recent decades, it is well recognised that the oral bioavailability of drug candidates is negatively influenced by trends in drug discovery for candidates with increasing molecular size, lipophilicity and hydrophobicity [1,2,3]. In addition, increasingly new drug candidates fall outside the classical Rule-of-Five drug discovery space, resulting in solubility and/or permeability challenges [2,3]. Such beyond Rule-of-Five drugs necessitate a bio-enabling formulation approach addressing both solubility and permeability limitations simultaneously. One formulation approach addressing both limitations is a lipid-based formulation (LBF). LBFs may improve oral bioavailability via a number of mechanisms including avoidance of a drug dissolution step, an increased extent of drug solubilisation in intestinal fluids, lipid excipient-mediated increased intestinal permeability as well as possible enhancement of lymphatic uptake from the gastrointestinal tract [4,5]. However, the LBF development is complicated by the fact that for many beyond Rule-of-Five drug substances, it is not possible to solubilise the dose in a relevant amount of lipid vehicle due to an inherent low drug solubility in lipid vehicles. For such challenging drug candidates, displaying both poor water and lipid solubility, advanced formulation strategies that increase drug loading in lipid vehicles, such as lipophilic salts/ionic liquids [6,7,8,9,10] or supersaturated LBFs (sLBF) [11,12,13], have recently been proposed.

sLBFs are lipid-based solutions that contain a drug concentration above the thermodynamic solubility of the drug in the lipid vehicle. Such supersaturated formulations are commonly prepared using heat [11,12,13]. While supersaturated formulations may display kinetic stability, thermodynamics dictate that precipitation will eventually occur depending on the drug properties, lipid vehicle, supersaturation ratio and storage conditions. sLBFs offer significant advantages in a pre-clinical drug product development setting, offering a balance between the potential to maximise oral exposure and facilitating ease of preparation and administration in pre-clinical models, which is particularly relevant in early toxicological drug evaluations [14]. In the literature, sLBFs of small and highly lipophilic drugs such as halofantrine [12], simvastatin [11], fenofibrate [15], cinnarizine [16] and R3040 [17] have been reported. In most cases, a higher oral bioavailability was observed in comparison to either an aqueous suspension, commercial product or an undersaturated lipid solution. While sLBFs have been successfully used for small molecules that are highly lipophilic and show low–moderate hydrophobicity, it remains unclear whether the formulation concept is also applicable to larger-molecular-weight (e.g., >500 g/mol) and/or moderate–high hydrophobic drug candidates. 

**Figure 1:**
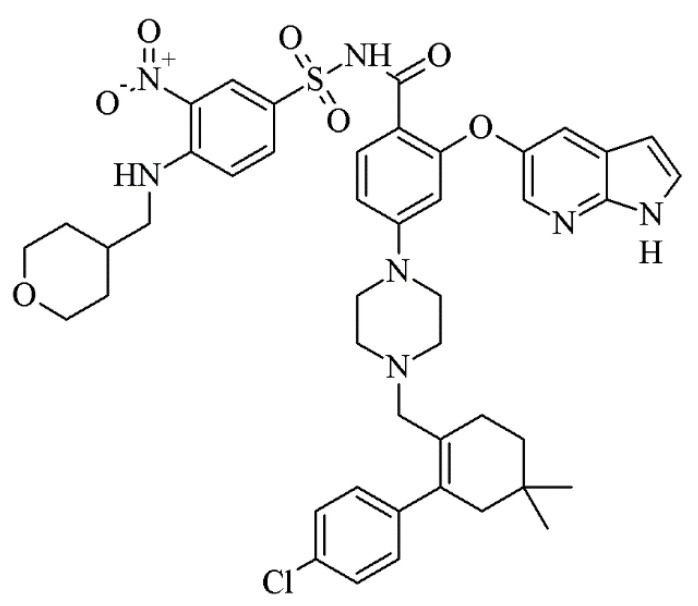
Chemical structure of venetoclax.

Venetoclax (Figure 1), a selective B-cell lymphoma-2 inhibitor licenced in 2016 for the treatment of leukaemia, displays a high lipophilicity (log*P* 5.5), high molecular weight (868.44 g/mol) [18] and a mid-range melting point (138 °C onset) [19]. It is classified as a BCS class IV [20] drug and exhibits a solubility in aqueous buffer of < 0.0042 µg/mL at pH 7.4 [18]. The commercialised formulation, Venclyxto^®^, exhibits a substantial food-dependent bioavailability with a 3.4-fold increase in oral bioavailability after a low-fat meal and a 5-fold increase after a high fat meal compared to the fasted state [18]. Venclyxto^®^ is considered to be a solid dispersion in copovidone [19] and must be taken with food to achieve an adequate bioavailability [19] suggesting that dietary lipids may be beneficial for improving oral absorption of venetoclax. Venetoclax was chosen as a model drug for the present study in order to explore whether it can be supersaturated in a lipid vehicle and whether a sLBF improves in vivo bioavailability using landrace pigs as animal model.

## 2. Materials and Methods 

**Materials.** Two venetoclax batches were purchased from Kemprotec Ltd. (UK) (batch 1: #1705150, batch 2: #180620). One further venetoclax batch was ordered from Sigma-Aldrich (Ireland) (batch 3: #11777). Olive oil, highly refined and low acidity, capric acid, L-α-phosphatidylcholine type XI-E (PC) (768 g/mol), taurodeoxycholic acid (NaTDC) and pancreatic lipase (8 × USP) were ordered from Sigma-Aldrich (Ireland). Capmul MCM^®^ and Captex^®^ 1000 were kindly donated by Abitec corporation. A sample of Peceol^®^ was kindly provided by Gattefossé (France) and SIF powder version 1 was kindly donated by biorelevant.com (UK). All other chemicals and solvents were of analytical or high-performance liquid chromatography (HPLC) grade and were purchased from Sigma-Aldrich (Ireland) and used as received.

**Differential scanning calorimetry (DSC).** The thermal behaviour of venetoclax was studied using a TA Q1000 with a TA Refrigerated Cooling System 90 (TA Instruments, New Castle, DE, USA). The cell was purged with nitrogen at 50 mL/min. The melting temperature was measured by modulated DSC. Venetoclax (5 mg) was weighed into a T-zero pan and heated from 20 to 200 °C at 3 °C/min. The modulation amplitude was set to ± 1.0 °C and the modulation time to 60 sec. 

The crystallisation behaviour of venetoclax was studied using the protocol by Baird et al. [21]. In brief, 2 mg venetoclax was weighed into a T-zero pan and heated to 165 °C at 10 °C/min, held isothermally for 3 min, cooled to −75 °C at 20 °C/min and reheated to 160 °C at 10 °C/min. As no crystallisation was observed during cooling and reheating, the heating rate was lowered to 2 °C/min. The experiments were run in triplicates. The glass-forming ability of drugs was categorised according to Baird et al. into class I (crystallisation during cooling prior to the glass transition temperature (*T_g_*)), class II (no crystallisation during cooling, but crystallisation was observed upon reheating above *T_g_*) and class III (no crystallisation observed during cooling nor reheating to its melting point) [21]. Additionally, the absence of crystals after heating and upon cooling was confirmed by hot-stage microscopy. 

**Hot-stage microscopy.** Samples were placed in a glass crucible onto a Linkam THMS 600 hot stage connected to a TP94 temperature controller (Linkam Scientific Instruments, Tadworth, UK). Samples were heated to 165 °C at 10 °C/min, held isothermally for 3 min, cooled to 25 °C at 20 °C/min and continuously monitored using an Olympus BX51 with an Olympus SC100 camera operated by Olympus Stream essentials 2.3.3. Images were collected at 40× magnification and the light was polarised using the polariser U-POT and analysed with the analyser U-ANT. The absence of crystals was assumed, if no birefringence was observed. The images are presented in the Supplementary Information (Figure S2).

**Cross-polarised light microscopy**. The absence of crystals in the supersaturated solution was confirmed by means of cross-polarised light microscopy using an Olympus BX51 with an Olympus SC100 camera operated by Olympus Stream essentials 2.3.3. The light was polarised using the polariser U-POT and analysed with the analyser U-ANT. The absence of crystals was assumed, if no birefringence was observed.

**Solubility studies.** The solubility was determined in olive oil, Captex^®^ 1000, Peceol^®^ and Capmul MCM^®^. Excess of venetoclax was added to 2 mL of the excipients and stirred at 200 rpm (25% power) (MIXdrive 15 HT, 2Mag AG, Germany) at 37 °C. Samples were taken after 24, 48, and 72 h. In the case of Peceol^®^ and Capmul MCM^®^, a high amount of venetoclax dissolved instantly upon addition to the excipient (exceeding the thermodynamic solubility), which lead to precipitation after 24–48 h. Therefore, Peceol^®^ and Capmul MCM^®^ samples were left at 37 °C up to 48 h before the first sample was taken. All samples were centrifuged at 21,380× *g* (Mikro 200 R, Hettich GmbH, Germany) and 37 °C for 15 min. The supernatant was transferred to a new sample tube and centrifuged again under identical conditions. To solubilise the oily excipient, the supernatant was diluted in acetonitrile, ethyl acetate (1:3, *v*/*v*), followed by further 1:10 (*v*/*v*) dilution with acetonitrile:ethyl acetate (3:1, *v*/*v*). The obtained samples were diluted appropriately with mobile phase before analysis by reverse phase HPLC as described below. All samples were run in triplicates.

**Biorelevant solubility.** Fasted state-simulated intestinal fluid (FaSSIF) and fed state-simulated intestinal fluid (FeSSIF) were prepared according to the instructions by biorelevant.com. Solutions of FeSSIF were prepared and used directly, whereas FaSSIF was left at room temperature for 2 h prior to usage. Excess of venetoclax was added to 2 mL FaSSIF and FeSSIF, respectively. The suspensions were placed in a water bath shaker with 200 shakes/min at 37 °C (GLS400, Grant Instruments, UK). Samples were taken at 3, 6 and 24 h and centrifuged at 21,380× *g* (Mikro 200 R, Hettich GmbH, Germany) for 15 min at 37 °C. The supernatant was transferred to a new sample tube and centrifuged again under identical conditions. Samples were diluted 1:10 (*v*/*v*) with mobile phase before analysis.

The samples were analysed using an Agilent 1200 series HPLC system (Agilent Technology Inc., Santa Clara, CA, US) that comprised a binary pump, degasser, column oven, autosampler and variable wavelength detector. Data analysis was performed with EZChrom Elite^®^ version 3.2. Venetoclax was separated from the sample matrix with a Zorbax^®^ Eclipse Plus-C18 column (5 μm, 4.6 mm × 150 mm) including a Zorbax^®^ Eclipse Plus-C18 guard column (5 μm, 4.6 mm × 12.5 mm) at 40 °C. The mobile phase consisted of (a) acetonitrile with 0.5% (*v*/*v*) trifluoroacetic acid (TFA) and (b) water with 0.5% (*v*/*v*) TFA at a ratio of 53:47 (a:b, *v*/*v*) and was used at a flow rate of 1 mL/min. The injection volume was 20 μL and the detection wavelength was set to 316 nm. The limit of detection (LOD) was 20 ng/mL and the limit of quantification (LOQ) was 65 ng/mL, determined using the standard error of y-intercept according to the International Council for Harmonisation (ICH) Q2 guideline [22].

**In vitro lipolysis: drug solubilisation during formulation dispersion and digestion.** In vitro lipolysis was performed using a pH-stat apparatus (Metrohm AG, Herisau, Switzerland), comprising a Titrando^®^ 907 stirrer, 804 Ti-stand, a pH electrode (Metrohm AG, Herisau, Switzerland) and two 800 Dosino^®^ dosing units coupled to a 20 mL automatic burette. The system was operated by the Tiamo^®^ 2.2 software. The in vitro protocol was amended from Williams et al. [23,24] as reported previously [25]. In brief, the buffer contained 2 mM TRIS maleate, 150 mM NaCl, and 1.4 mM CaCl_2_ 2H_2_O, adjusted to pH 6.5. For the digestion experiments, the buffer was supplemented with 3 mM NaTDC and 0.75 mM PC (digestion buffer) and stirred for 12 h before further usage. The pancreatin extract was prepared freshly by adding 5 mL of 5 °C digestion buffer to 1 g of porcine pancreatic enzymes (8x USP), which was vortexed thoroughly. The mixture was centrifuged for 15 min at 5 °C, 2800× *g* (Rotina 380 R, Hettich GmbH, Germany) and 4 mL of supernatant was recovered and stored on ice before further usage. 

For the in vitro lipolysis experiment 1.583 g of lipid formulation was dispersed into 57 mL of digestion buffer for 10 min. Three 1 mL samples were taken at 2.5, 5 and 10 min from the middle of the vessel. pH of the media was adjusted and maintained at 6.5 using 0.2 M NaOH. To the remaining 54 mL (1.5 g lipid formulation) dispersion 6 mL of pancreatin extract was added to initialise digestion. After 60 min the released non-ionised free fatty acids (FFAs) were determined by a pH increase in the buffer to pH 9. An additional blank titration using the digestion buffer was performed and the released mmol of FFAs from the blank was subtracted from the mmol of FFAs released from the surfactant formulations

Samples of 4.9 mL were taken at 5, 10, 15, 30, 45 and 60 min during the digestion experiment from the middle of the vessel. In each sample and after 60 min the enzymes were inhibited by the addition of 1 M 4- Bromophenylboronic acid in methanol (5 μL per mL sample). All samples containing a lipid phase were centrifuged at 37 °C and 400,000× *g* for 30 min (Beckman Coulter Optima L-90K, Rotor: VTI 65.2). Samples, that did not contain a lipid phase (aqueous suspension) were centrifuged at 37 °C and 21,000× *g* for 30 min using a benchtop centrifuge (Micro 200 R, Hettich GmbH, Germany). The lipid phase was dissolved in a mixture of ethyl acetate and acetonitrile (3:1 *v*/*v*) and diluted with 1:10 (*v*/*v*) with a mixture of ethyl acetate and acetonitrile (1:3 *v*/*v*). The solid phase was added to 2 mL of a mixture of ethyl acetate and acetonitrile (3:7 *v*/*v*) and ultrasonicated for 10 min. The aqueous phase was diluted 1:10 (*v*/*v*) with mobile phase. The resulting lipid, solid and aqueous phase samples were subsequently centrifuged at 37 °C and 21,000× *g* for 30 min. The resulting supernatants were diluted further with mobile phase and analysed by HPLC as stated above.

The digestibility of Peceol^®^ was calculated as previously described [26] using the theoretical released FFAs per g of Peceol^®^ utilising the saponification value (166 mg) on the certificate of analysis:

(1) $$\mathrm{Theoretical}\;\mathrm{FFAs}\;\left[\mathrm{mmol}\right]=\frac{\mathrm{SV}\;\left[\mathrm{mg}\right]}{56.1056\;\left[\frac{g}{\mathrm{mol}}\right]},$$

where theoretical FFAs are the maximal amount of FFAs that can theoretically be released from Peceol^®^ in mmol per g of excipient, SV the saponification value in mg KOH per g of excipient and 56.1056 g/mol the molecular weight of KOH. The maximal absolute amount of theoretically released FFAs can be calculated by multiplying by the amount of excipient used (i.e., 1.5 g in this study). The % digested can be calculated as follows:

(2) $$\%\;\mathrm{digested}=\frac{\mathrm{Released}\;\mathrm{FFAs}\;\left[\mathrm{mmol}\right]}{\mathrm{Theoretical}\;\mathrm{FFAs}\;\left[\mathrm{mmol}\right]}\times 100\%,$$

where the released FFAs provide the total amount that is released at the end of the digestion experiment including the non-ionised FFAs determined by raising the pH to 9.0 and the theoretical FFAs were calculated using Equation (1).

**Formulation development of supersaturated LBFs.** The increase in venetoclax concentration above its solubility in the excipients was achieved by incorporating venetoclax at an elevated temperature, i.e., the formulations were heated, kept at elevated (isothermal) temperature and cooled to room temperature. In detail, excess venetoclax was added to Peceol^®^, olive oil, Capmul MCM^®^, Capex^®^ 1000, respectively, and suspended at 600 rpm (Stuart CD 162 heat-stir, Cole-Parmer, UK). The vial was sealed with parafilm and a continuous nitrogen stream was purged through the vial to prevent any oxidation during the heating and cooling process. While continuously stirring at 600 rpm, the suspensions were heated to 70 °C and kept at 70 °C for 10 min (Stuart CD 162 heat-stir, Cole-Parmer, UK) followed by cooling to 23 ± 2 °C by elevating the vial from the hot plate. Once the mixtures reached a temperature below 25 °C, a 0.5 mL sample was taken, and the remaining mixture was heated and cooled again using the same protocol described above. After this second cycle, a second 0.5 mL sample was taken, and the mixture was heated and cooled a third time using the same conditions as described above followed by a final 0.5 mL sample. During all heating and cooling cycles, excess venetoclax was present to investigate the maximum achievable concentration with this protocol. The samples were centrifuged at 21,380× *g* and 37 °C (Mikro 200 R, Hettich GmbH, Germany) for 15 min. The supernatant was transferred to a new sample tube and centrifuged again using identical conditions. The supernatant was used for HPLC analysis using the same dilutions and solvents as described for the solubility studies in the lipid excipients above. The remaining suspension after the third heating cycle was centrifuged under identical conditions as stated above and transferred to a new sample tube for stability testing and stored at room temperature. The stability of promising formulations was evaluated using polarised light (Allen Viewer Type LV28, P.W. Allen & Co Ltd., Tewkesbury, UK). Due to the high drug amount needed, these formulations and stability tests were conducted once.

**Formulations for in vivo and in vitro studies.** For the in vivo used powder venetoclax formulation, 100 mg venetoclax were weighed into hard gelatine capsules of size 00. For the in vivo used Peceol^®^ suspension, 50 mg venetoclax was weight into hard gelatine capsules of size 000 and 1 mL of Peceol^®^ (melted at 50 °C and cooled to 37 °C) was added. Venetoclax was dispersed by vigorous shaking. 

In the in vitro experiments, the powder formulation was resembled by an aqueous suspension. The aqueous suspension was prepared by adding 50 mg venetoclax to 1 mL water followed by ultrasonication. The suspensions were stirred constantly to prevent sedimentation before usage. The Peceol^®^ suspension was prepared by melting Peceol^®^ at 50 °C and cooling it to 37 °C before adding 50 mg venetoclax to 1 mL Peceol^®^ followed by an over-night stir. 

The supersaturated lipid solution was prepared by adding 300 mg venetoclax to 6 mL Peceol^®^ (50 mg/mL). The mixture was stirred at 600 rpm (Stuart CD 162 heat-stir, Cole-Parmer, UK) and sealed with parafilm. A continuous nitrogen stream into the vial removed oxygen throughout the manufacturing process. After suspending the drug particles, the obtained suspension was slowly heated to 70 °C (Stuart CD 162 heat-stir, Cole-Parmer, UK). The mixture was kept at 70 °C for 10 min and cooled to 25 °C while continuously stirring at 600 rpm. Subsequently, the mixture was heated a second time under the same conditions as stated above and cooled to room temperature. The absence of crystals was confirmed using cross-polarised light microscopy. For the in vivo study, sLBF was administered in hard gelatine capsules of size 000 (1 mL per capsule).

**In vivo study**. All experiments were approved and conducted with licences issued by the Health Product Regulatory Authority, Ireland (project licence AE19130/P058) as directed by the EU Statutory instruments of the EU directive 2010/63/EU (Protection of Animals used for Scientific Purposes). Local ethical approval was granted by University College Cork Animal Experimentation Ethics Committee (AEEC). A randomised, three-way cross-over study in 3 male landrace pigs (15–17 kg) was conducted and each pig received a single dose of 100 mg venetoclax. Pigs were fed approximately 175 g of standard weanling pig pellet feed twice daily. In the fasted study legs, the final feed of 175 g was given 24 h prior to dosing. As part of the study design, any remaining food was removed 16 h before dosing. However, no food remained at this point in any of the groups. On day 1 of the experiment, an intravenous catheter was inserted from the ear vein into the jugular vein under general anaesthesia, which was used for blood sampling throughout the study. On day 3, following an overnight fast of 16 h, pigs were administered either a reference capsule with venetoclax powder or two capsules of either venetoclax Peceol^®^ suspension or a venetoclax supersaturated lipid solution (sLBF) with the aid of a dosing gun followed by 50 mL of water via a syringe. In order to control the water intake with the dosage forms, the water availability was restricted for 3 h post-dosing. At all other times, water was available ad libitum. To facilitate handling during the oral administration, an intramuscular dose of ketamine (5 mg/kg) and xylazine (1 mg/kg) was administered in both studies. Blood samples were collected after 0.5, 1, 1.5, 2, 3, 4, 5, 6, 7, 8, 9, 10, 12, and 24 h in heparinised tubes. Upon collection, blood samples were immediately centrifuged at 3000× *g*, 4 °C for 5.5 min (Eppendorf 5702 R, Rotor A-4-38, Eppendorf Ltd., Stevenage, UK). The supernatant plasma was harvested and stored at −20 °C until further analysis. A 6 day washout period was maintained between the study legs. Due to a rupture and spillage of one sLBF capsule during oral dosing to one of the pigs, this experimental unit was excluded, resulting in n = 2 for the sLBF study group.

**Bioanalysis.** The plasma concentrations of venetoclax were determined by reversed phase HPLC. The Agilent 1260 series HPLC system (Agilent Technology Inc., Santa Clara, CA, US) comprised a binary pump, degasser, temperature controlled autosampler, column oven and diode array detector. The system was operated, and the data analysed with EZChrom Elite^®^ version 3.3.2. A Zorbax^®^ Eclipse Plus-C18 column (5 μm, 4.6 mm × 150 mm) with a Zorbax^®^ Eclipse Plus-C18 guard column (5 μm, 4.6 mm × 12.5 mm) was used for the separation of venetoclax. The mobile phase consisted of water and acetonitrile with 0.5% (*v*/*v*) TFA at a ratio of 47:53 (*v*/*v*) and was used at a flow rate of 1.0 mL/min. The sample and column temperature were set at 5 °C and 40 °C, respectively, and the detection wavelength was set to 250, 290 and 316 nm. Venetoclax was extracted from the plasma samples by liquid-liquid extraction. To 500 μL of pig plasma, 50 μL vemurafenib (internal standard dissolved in acetonitrile) and 450 μL acetonitrile was added. The mixture was mixed thoroughly, and 1 mL of ethyl acetate was added followed by mixing for 15 s. The mixture was centrifuged at 25 °C, 11,500× *g* for 5 min (Mikro 200 R, Andreas Hettich GmbH & Co. KG, Germany). The supernatant (1.5 mL) was recovered and transferred to a new sample tube. Subsequently, the supernatant was dried at 60 °C under a nitrogen stream. To the remaining plasma sample, 1 mL of ethyl acetate was added and the mixture was thoroughly mixed and centrifuged using the same condition as above. The supernatant was recovered and transferred to the same sample tube that contained the supernatant of the plasma sample after the first centrifugation (which was dried as described above). After drying of the supernatant, the residues were reconstituted in 100 μL mobile phase (excluding TFA), followed by centrifugation at 25 °C, 11,500× *g* for 5 min (Mikro 200 R, Andreas Hettich GmbH & Co. KG, Germany). The injection volume used for HPLC analysis of the supernatant was 50 μL. The LOD and LOQ in plasma by this method were 6 and 20 ng/mL, respectively, determined using the standard error of y-intercept according to ICH Q2 guideline [22]. Linearity was confirmed between 25 and 2.5 μg/mL.

**Data analysis.** A one-way analysis of variance (one-way ANOVA) was performed for the lipolysis data using Tukey’s post-hoc test to compare the different formulation performances and Bartlett’s test to check for equal variance. The pharmacokinetic parameters were calculated using Gastro Plus^®^ version 9.5 (Simulations Plus Inc., Lancaster, CA, US) and in-house intravenous data. The plasma concentration profiles were analysed by non-compartmental analysis. 

The statistical analysis for all in vivo parameters was performed using a one-way ANOVA after using Bartlett’s test to check for equal variance. The pairwise comparison of the groups was based on Tukey’s multiple range test. All statistical analyses were calculated using GraphPad Prism^®^ 5 (GraphPad Software Inc., San Diego, CA, US).

## 3. Results

### 3.1. Biorelevant Solubility

Venetoclax is a structural bulky and high-molecular-weight drug, displaying a mid-range melting point and high log*P* (Table 1, Figure 1, Figure S1). Initially, three separate batches of the drug substance were sourced in order to assess the inter-batch variability of in vitro characteristics. Venetoclax solubility for each batch was measured in FaSSIF and FeSSIF at 37 °C. The results are presented in Table 2.

The solubility values in FaSSIF were similar for all studied batches, with approximately 1.4–1.7% drug dissolved of a 100 mg dose in 250 mL of gastrointestinal fluid. In the fed-state media, the solubility increased 4.8-fold to approximately 6.6–7.7% of a 100 mg dose in 250 mL of gastrointestinal fluid, which was in line with the in vivo food effect reported for venetoclax (Table 1). In contrast to the atypical solubility behaviour of venetoclax in lipid vehicles (i.e., high inter-batch variability and oversaturation), venetoclax solubility in biorelevant media was similar between the three batches. This most likely reflects the ability to more quickly achieve equilibrium solubility in aqueous buffers due to a lower viscosity, higher wettability and the presence of bile salts/phospholipids to aid solubilisation.

### 3.2. Solubility in Lipid Excipients

In addition to biorelevant solubility, the solubilities of the three venetoclax batches were determined in olive oil, Captex^®^ 1000, Peceol^®^ and Capmul MCM^®^, respectively, at 37 °C. Venetoclax solubility in lipid excipients is shown in Table 3. 

**Figure 2:**
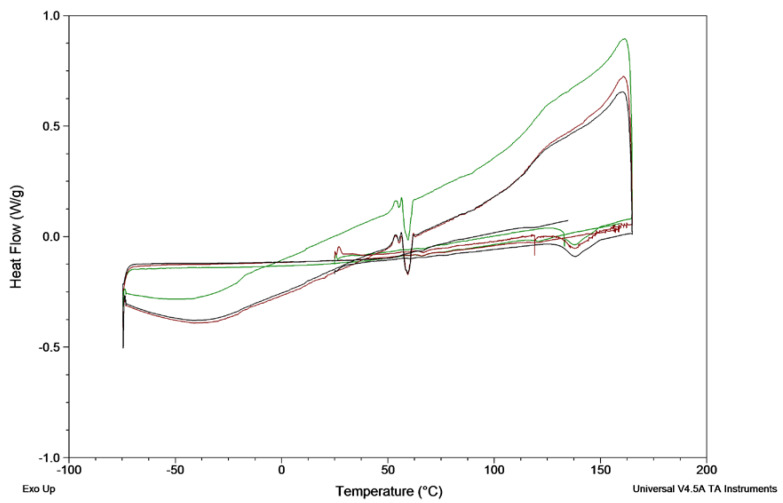
DSC of venetoclax batch 1 (1705150). A heat–cool–heat protocol was used to determine the glass-forming ability (n = 3).

Variability in venetoclax solubility in the lipid vehicles between the three tested venetoclax batches was observed. The solubility of different batches of venetoclax in Peceol^®^ varied 6.7-fold from 2.9 ± 0.2 mg/mL to 19.4 ± 2.0 mg/mL. For olive oil, a variation of 9.2-fold, for Capmul MCM^®^ a 1.6-fold variation and for Captex^®^ 1000 a 2.3-fold variation was observed across the different venetoclax batches. While two venetoclax batches showed a comparable solubility within an approximately 2-fold difference (batch 2 and 3), batch 1 was considerably different in the case of both long chain excipients Peceol^®^ and olive oil. Despite the variability, the solubility screening in pure lipid excipient indicated that venetoclax showed a higher solubility in monoglycerides than triglycerides. Additionally, a dose loading comparable to the marketed product of 100 mg/tablet was not reached in any of the studied excipients precluding the use of a classical lipid solution approach, i.e., not a supersaturated and hence stable system, for venetoclax in these lipid vehicles. It became apparent during solubility screening of venetoclax in monoglyceride vehicles (i.e., Peceol^®^ and Capmul MCM^®^) at 37 °C, that the drug appeared to spontaneously oversaturate following addition of excess drug to the lipid, which visually appeared to dissolve. However, over time, venetoclax precipitated and thermodynamic equilibrium was achieved. This phenomenon was also demonstrated quantitatively, where higher drug concentrations were observed in the first 24–48 h, compared to concentrations at later time points. In both the monoglyceride vehicles, this oversaturated state was stable for up to 48 h. Such atypical behaviour of venetoclax displayed challenges for the methodology of determining the thermodynamic solubility of venetoclax in the lipid vehicles. In fact, while drug solubility at 37 °C was monitored for up to 72 h post-precipitation, the solubility determined in the lipid excipients may not reflect true ‘thermodynamic’ equilibrium, but rather a higher energy intermediate state. As a result of this behaviour, where excess drug appeared to dissolve initially and precipitated over 12–48 h in the solubility studies presented in Table 3, the first concentrations were only determined (i.e., first sampling point t = 24 h) after the drug had precipitated from an oversaturated system. It is likely that thermodynamic equilibrium would be achieved over an extended storage period (i.e., weeks). However, in this study with a 72 h study period, equilibrium solubility may not have been achieved, hence apparent solubilities are reported. The thermodynamic equilibrium may have been further influence by the viscosity of the lipid excipients and the glass-forming ability of venetoclax (class III, Table 1, Figure 2). Nevertheless, this venetoclax-specific effect indicated that monoglycerides may be a good solubilising vehicle to promote and maintain drug supersaturation. 

### 3.3. Thermal Properties of Venetoclax

In order to further investigate the atypical behaviour of venetoclax, the thermal behaviour of the different batches was studied. The results are presented in Table 4 and Figure S1 and showed that venetoclax batch 1 had a melting point of 138.6 ± 0.1 °C, which was similar to the reported onset of the melting point of 138 °C (Table 1) [19], an enthalpy of fusion of 19.9 ± 0.7 kJ/mol and entropy of fusion of 4.8 ± 0.2 × 10^−2^ kJ/mol/K. Batch 2 showed similar results, with a melting point of 140.2 ± 0.1 °C, entropy of fusion of 18.4 ± 0.4 kJ/mol and an entropy of fusion of 4.5 ± 0.1 × 10^−2^ kJ/mol/K. However, batch 3 showed two endothermal events, with one major peak and one minor peak, indicating that several crystalline structures could be present. While the major peak showed a similar melting point compared to the other batches, the entropy and enthalpy of fusion was different when compared to batch 1 and 2. While batch 3 showed a distinct different thermal behaviour compared to batch 1 and 2, the solubility of batch 3 was similar to the solubility of batch 2 and different to batch 1. 

In addition, the glass-forming ability of venetoclax batch 1 was determined by DSC. The results are presented in Figure 2 and Table 1. The first heating cycle confirmed the melting point of venetoclax batch 1 as described above. In the second cycle, the venetoclax melt was cooled at a rate of 20 °C/min in order to generate amorphous venetoclax. No crystallisation event was observed during cooling. Hot-stage cross-polarised microscopy confirmed the generation of amorphous venetoclax and revealed no crystallisation during cooling to room temperature (25 °C) (Figure S2). In the reheating cycle, no endothermic peak was present showing that venetoclax did not tend to recrystallise, categorising venetoclax as a class III glass former according to Baird and co-workers definition [21].

### 3.4. Supersaturation of Venetoclax in Lipid Excipients

**Figure 3:**
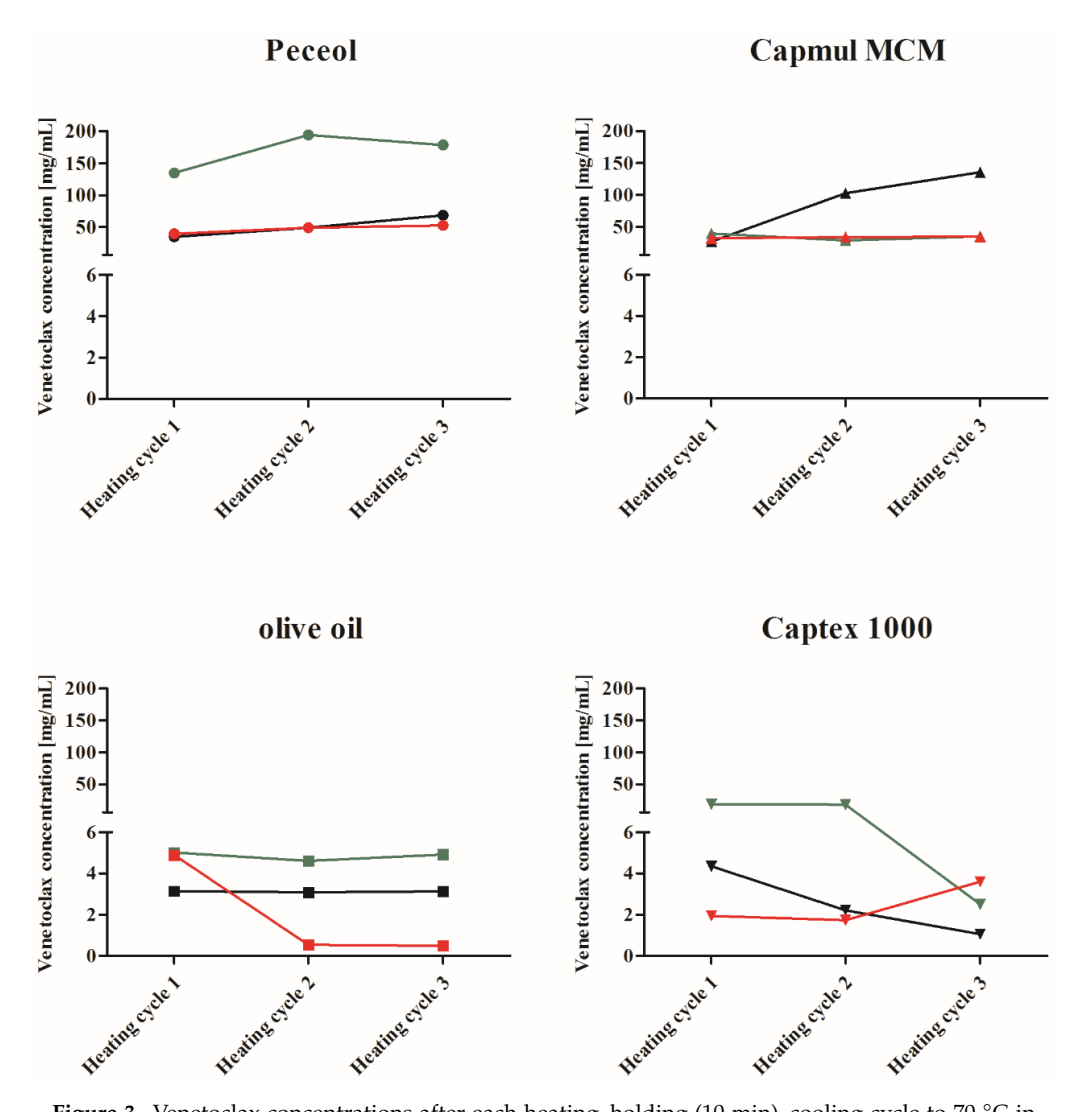
Venetoclax concentrations after each heating–holding (10 min)–cooling cycle to 70 °C in Peceol^®^ (●), Capmul MCM^®^ (▲), olive oil (■) and Captex^®^ 1000 (▼). Batch 1 (black), batch 2 (green) and batch 3 (red), where n = 1 for initial screening.

Applying a temperature-induced supersaturation approach, suspensions of venetoclax in lipid excipients were heated to 70 °C, kept at 70 °C for 10 min followed by cooling to 25 °C. This protocol was repeated up to three times. An excess of drug (i.e., a solid phase) was present at all times during heating and cooling. The incorporated concentration of venetoclax, i.e., kinetic solubility, was measured after cooling in olive oil, Captex^®^ 1000, Peceol^®^ and Capmul MCM^®^, respectively. At the end of the experiment, the samples were centrifuged, and the supernatants of promising formulations were stored at room temperature to evaluate the stability of the supersaturated solutions. Due to the large quantities of venetoclax required in this screening study, an n = 1 was used at each sampling timepoint. The venetoclax concentration for all three batches in Peceol^®^, Capmul MCM^®^, olive oil and Captex^®^ 1000 is shown in Figure 3. The fold difference between the solubility at 37 °C and the maximum achieved concentration at 70 °C is shown in Table 5.

In all excipients, relatively higher concentrations were reached after the heating cycles compared to the solubility at 37 °C. While in the case of olive oil and Captex^®^ 1000 the concentration increases were high, ranging from 1.3- to 12.5-fold for olive oil and 3.7- to 28.2-fold in the case of Captex^®^ 1000, the total dissolved amount was in most cases below 5 mg/mL, which is insufficient relative to the clinical dose and precludes the use of triglycerides for the sLBF approach. For Capmul MCM^®^, venetoclax concentrations increased between 5.9- and 22.3-fold. However, only batch 1 was able to generate venetoclax concentrations above 50 mg/mL. Additionally, Capmul MCM^®^ showed a poor stability of <1 day, limiting the use of this sLBF to possible ad hoc preparation in pre-clinical development. In the case of Peceol^®^, a concentration > 50 mg/mL was achieved with all studied batches, resulting in an increase in venetoclax concentration of 3.5- to 67.9-fold in the supersaturated state. Interestingly, for Peceol^®^, the supersaturated concentration of venetoclax was maintained in the formulation for in excess of 2 weeks. 

The consecutive heating cycles were designed to investigate whether further thermal exposure would increase venetoclax concentrations in the excipients. In the case of Peceol^®^, venetoclax concentrations increased, while in the case of Capmul MCM^®^, an increase was observed in only one case (batch 1), with the other batches showing a similar venetoclax concentration throughout the heating cycles. Interestingly, in the case of triglycerides, venetoclax concentration decreased in some cases upon each heating cycle. The initial supersaturated Captex^®^ 1000 showed a decrease in the amount of venetoclax with each further heating cycle for batch 1 and 2. A similar decrease was observed for batch 3 in olive oil with the second heating cycle. 

As a result of these initial screening trials, a Peceol^®^ sLBF was used for further in vitro and in vivo evaluation due to the high and maintained concentrations of drug in the vehicle over 2 weeks. The drug amount in the formulation was fixed at 50 mg/mL, which was below the maximum achievable concentration in Peceol^®^ (batch 1: 68.3 mg/mL, batch 2: 178.62 mg/mL, and batch 3: 52.4 mg/mL). Batch 1 was chosen for further studies, based on displaying the lowest fold increase in supersaturation at 50 mg/mL (i.e., 2.5-fold) and hence a lower risk of precipitation relative to batch 2 (17.5-fold) and batch 3 (11.6-fold). The sLBF was produced using two heating cycles rather than three to reduce the thermal exposure to drug and excipient. Short-term storage studies with sLBFs produced under these conditions confirmed that the concentration of venetoclax in the Peceol^®^ sLBF (batch 1) was maintained at 50 mg/mL for up to 20 days with no macroscopic signs of instability.

### 3.5. Drug Solubilisation during In Vitro Dispersionand Digestion

**Figure 4:**
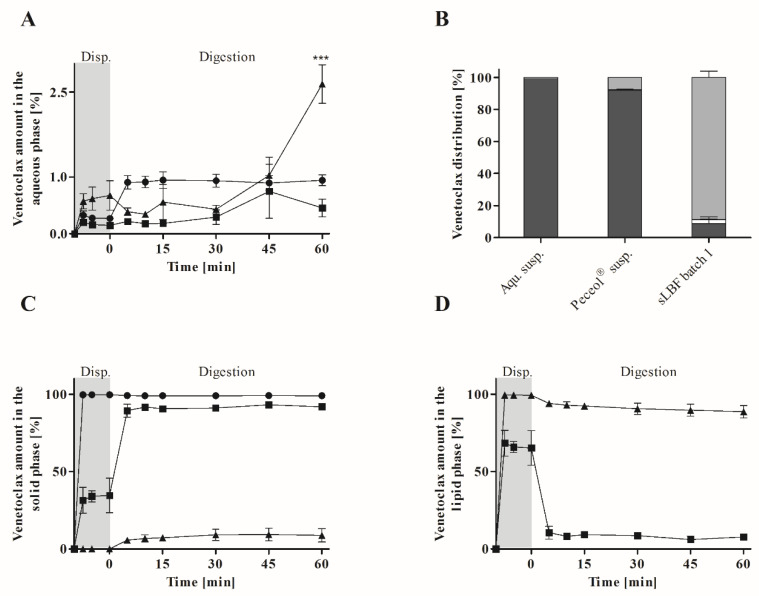
In vitro lipolysis of venetoclax formulated as an aqueous suspension (5% *w*/*v*) (●), a Peceol^®^ suspension (5% *w*/*v*) (■), and a supersaturated Peceol^®^ solution (sLBF) (5% *w*/*v*) (▲). (**A**): % of venetoclax in the aqueous phase. Statistical significance is compared to Peceol^®^ and the aqueous suspension. (**B**): Distribution of venetoclax into the different phases after 60 min of lipolysis. (**C**): % of venetoclax in the solid phase. (**D**): % of the venetoclax in the lipid phase. All experiments were run with n = 3 and results are shown as the mean ± SD. Statistical difference is indicated as ***, where *p* ≤ 0.001.

In order to study how digestion affects the supersaturated formulation, sLBF (50 mg/mL venetoclax supersaturated in Peceol^®^, 5% *w*/*v*) was assessed using the dynamic in vitro lipolysis model and compared to a Peceol^®^ suspension (50 mg/mL venetoclax mixed in Peceol^®^, 5% *w*/*v*) and an aqueous suspension (5% *w*/*v*). Initially, lipid formulations were dispersed in biorelevant buffer representing the fasted state for 10 min prior to initiation of the digestion by the addition of porcine pancreatic lipase. The recovery of venetoclax in the aqueous phase, lipid phase and solid phase during dispersion and digestion is shown in Figure 4.

**Figure 5:**
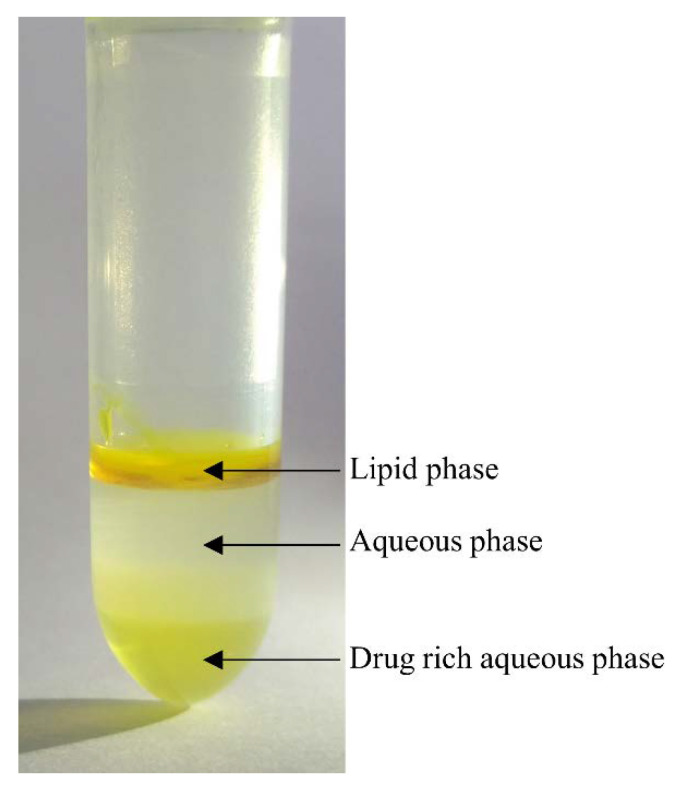
In vitro lipolysis sample of sLBF after ultracentrifugation. Four phases were present: lipid phase, aqueous phase, solid phase (not shown) and drug-rich aqueous phase.

The amount of venetoclax recovered in the aqueous phase is shown in Figure 4A. Upon dispersion and after 60 min of digestion, the highest venetoclax concentration in the aqueous phase was observed for sLBF. However, upon initiation of digestion, the sLBF showed a decrease in venetoclax concentration in the aqueous phase, which correlated well with the slow increase in the solid phase (Figure 4C). From 30 min of digestion onwards, the venetoclax concentration in the aqueous phase for sLBF started to increase from 0.4 ± 0.1% to 2.6 ± 0.3% of the added venetoclax dose, which was significantly different 60 min after initiation of digestion to all other formulations (*p* < 0.05). The time delay of 30 min to increase the venetoclax concentration in the aqueous phase suggested that digestion of the sLBF may be a key contributor to the high concentration of venetoclax at the end of the in vitro lipolysis. Additionally to the aqueous phase, for sLBF, a drug-rich aqueous phase was present after ultracentrifugation during digestion (Figure 5). In the case of the aqueous suspension, upon dispersion a lower venetoclax concentration was observed in the aqueous phase when compared to sLBF. Interestingly, upon initiation of digestion an approximately 3-fold increase in venetoclax concentration in the aqueous phase was observed for the aqueous suspension. This concentration of 0.9 ± 0.1% was maintained throughout digestion. The overall lowest concentration was observed for the Peceol^®^ suspension throughout dispersion and digestion, which resulted in 0.5 ± 0.2% of the venetoclax dose solubilised after 60 min of digestion. 

The amount of venetoclax in the lipid phase for sLBF was significantly higher compared to the amount of venetoclax in the solid and aqueous phase (*p* < 0.05), as presented in Figure 4D. The lipid phase contains mainly undigested or partially digested Peceol^®^, which contains mainly monoglycerides (44.9%), diglycerides (41.9%) and triglycerides (9.3%). In the employed in vitro lipolysis, 23.5 ± 0.9% of Peceol^®^ was digested after 60 min of digestion (according to equation 1 and 2). On average, 99.2 ± 0.2% of the venetoclax dose resided within the lipid phase during dispersion. The venetoclax dose in the lipid phase was considered absorbable, as the drug can partition into the aqueous phase, be absorbed through the lipid absorption pathway and be released by digestion of the lipid phase. The lipid phase of the sLBF demonstrated only an 8% decrease in venetoclax concentration over 60 min of digestion in the lipolysis experiment. In the case of the Peceol^®^ suspension, the venetoclax amount in the lipid phase was higher during dispersion compared to the solid and aqueous phase. The amount of venetoclax decreased from 66.5 ± 7.3% upon dispersion to 8.3 ± 2.3% during digestion, which correlated with the amount recovered in the solid phase (Figure 4C). This indicated that the venetoclax crystals in the Peceol^®^ suspension were entrapped in the lipid excipient, an observation which was similar to that previously demonstrated for lipid nilotinib suspensions [25]. Overall, a comparison between the formulations suggested that the sLBF had the ability to maintain higher concentrations in the lipid and aqueous phase relative to the suspensions, and hence may offer benefits in vivo.

Venetoclax recovery in the solid phase is presented in Figure 4C. The drug particles recovered in the solid phase need to undergo dissolution prior to absorption [27]. High amounts indicate that the formulation performance in vivo might be hampered by the poor dissolution and solubility of venetoclax in the aqueous media. The lowest amount of solid was recovered for the sLBF throughout dispersion and digestion. During dispersion, no solid phase was present. After initiation of digestion, drug precipitation occurred with up to approx. 9% of the theoretical dose recovered in the solid phase. Thus, the amount of drug that needed to undergo redissolution was the lowest for the sLBF suggesting a promising formulation performance in vivo. The highest amount of drug in the solid phase was recovered in the case of the aqueous suspension. Above 98% was recovered throughout dispersion and digestion in the solid phase, which was 11-fold higher compared to sLBF at the end of digestion. Upon dispersion, 33.3 ± 7.3% of the theoretical venetoclax dose was recovered in the solid phase for the Peceol^®^ suspension, which further increased to 89.4 ± 4.1% upon initiation of digestion, which was 10-fold higher compared to the sLBF. These results indicated that the suspended particles in the Peceol^®^ suspension were residing/entrapped in the lipid vehicle.

### 3.6. In Vivo Bioavailability of Venetoclax

**Figure 6:**
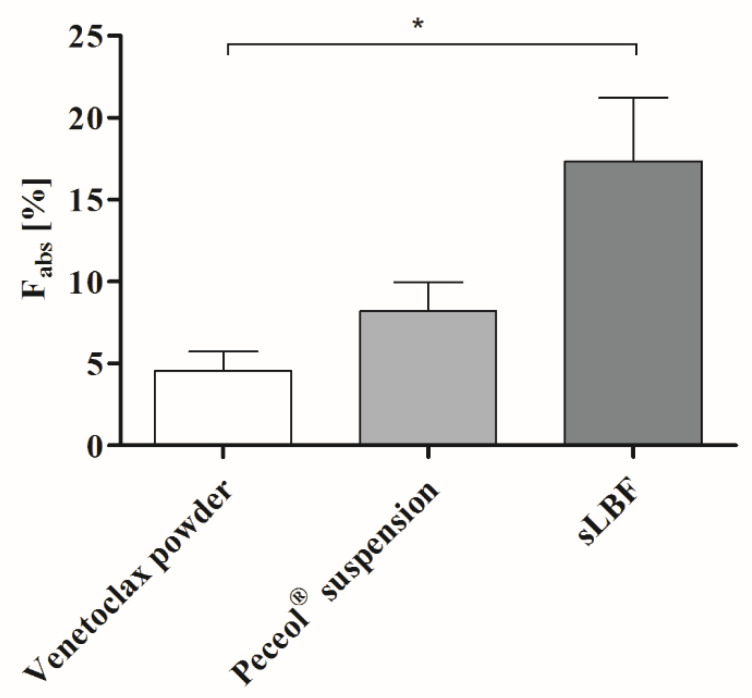
Absolute bioavailability (F_abs_) of 100 mg venetoclax as a venetoclax powder capsule, a Peceol^®^ suspension, and a supersaturated Peceol^®^ solution (sLBF) in male landrace pigs. All data is presented as the mean ± SD, where n = 3 (except for sLBF, where n = 2). Statistical difference is indicated as *, where *p* ≤ 0.05.

**Figure 7:**
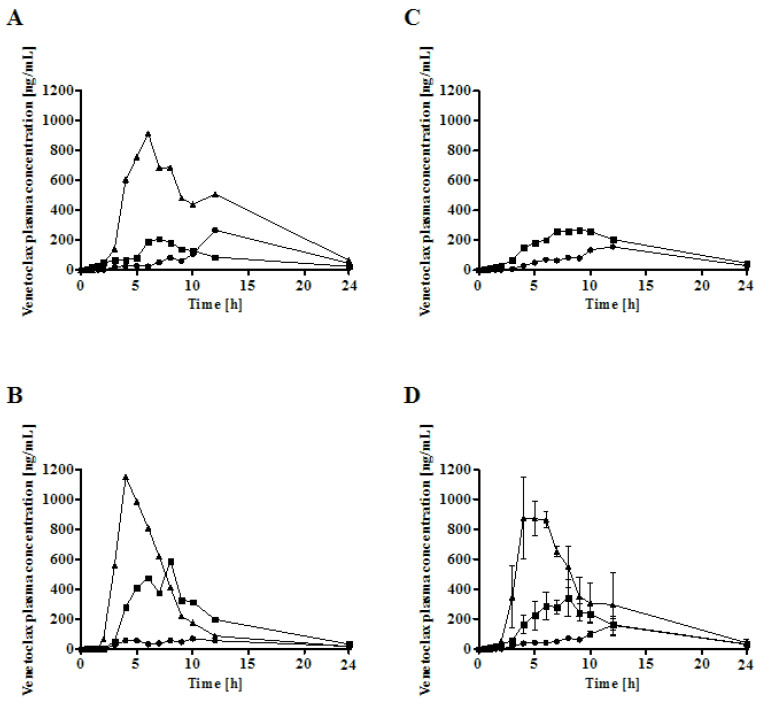
Venetoclax plasma concentration versus time profiles from 0 to 24 h for 100 mg venetoclax in male landrace pigs. Venetoclax powder capsule (●), Peceol^®^ suspension (5% *w*/*v*) (■), and sLBF (5% *w*/*v*) (▲). (**A**–**C**): Individual plasma concentration versus time profiles. (**D**): Mean plasma concentration versus time profiles.

The in vivo performance of venetoclax was evaluated in male landrace pigs. Each pig was administered 100 mg venetoclax as either a Peceol^®^ sLBF (50 mg/mL, 5% *w*/*v*)), Peceol^®^ suspension (5% *w*/*v*) or as a pure powder in a capsule. Absolute bioavailability was calculated using in-house data. The absolute bioavailability and the plasma concentration-time profiles are shown in Figure 6 and Figure 7, respectively, and the pharmacokinetic parameters in Table 6.

All lipid formulations showed a higher bioavailability when compared to the venetoclax powder capsule. The highest exposure was observed for sLBF with a relative bioavailability of 429.5 ± 151.1% compared to the venetoclax powder (Table 6, Figure 6). While there was a trend towards a higher exposure of sLBF compared to the Peceol^®^ suspension, sLBF was only significantly different from the venetoclax powder (*p* < 0.05). These results indicated that the formulation success depended on the lipid excipient and the solubilisation of the drug in the sLBF.

The in vivo study also demonstrated a faster absorption of venetoclax from sLBF. Relative to the powder capsule, sLBF demonstrated a statistically significant shorter time to reach the maximum plasma concentration (*t*_max_), mean residence time (MRT) and mean absorption time (MAT). Approximately 6 h shorter residence and absorption times were observed for the sLBF in comparison to the powder capsule. Relative to the Peceol^®^ suspension, sLBF showed a trend towards a quicker absorption and shorter MRT and *t*_max_. While this trend was not statistically significant, it indicated that the fast absorption, and hence less risk of precipitation, may be a factor in explaining the improved bioavailability for sLBF.

## 4. Discussion

LBFs represent a formulation approach for enhancing oral absorption of poorly water-soluble drugs [28] through biopharmaceutical benefits such as avoidance of a dissolution step and improved solubilisation in vivo upon dispersion and digestion of the formulation [4]. Further advantages may come from improved intestinal permeability and promotion of lymphatic transport. Many of these benefits have been demonstrated for highly lipophilic grease ball drugs, which are able to form a LBF solution. However, the drive of discovery technologies and methodologies such as high-throughput screening, the addition of lipophilic substituents during lead optimisation or the shift in therapeutic target identification increasingly produces more hydrophobic as well as lipophilic candidates with a trend of increased molecular weight [1]. A consequence of these particular hydrophobic drugs is an overall lower aqueous and lipid solubility, which may come with lower permeability due to molecular bulkiness and, therefore, the applicability of the classical LBF solutions is limited. As a consequence, strategies such as lipophilic salts/ionic liquids [9,10,29], supersaturated lipid solutions [11,12,13,15,17] or hybrid systems [30,31] were proposed that focus on increasing the drug load in LBFs. Nevertheless, it remains unclear whether LBFs offer biopharmaceutical benefits to enhance the bioavailability of these beyond Rule-of-Five drugs which display high lipophilicity and molecular weight as well as moderate hydrophobicity. 

Thermally induced supersaturation of venetoclax was observed in all lipid vehicles, with concentrations increasing between 26% and 6690% over the experimentally determined apparent solubility at 37 °C. In effect, heating the drug in lipid excipients, therefore, potentially increases drug loadings in lipid vehicles between 126% and 6790%. The % increases in drug loading in lipids observed for venetoclax were higher compared to typical dose loadings reported for other drugs in sLBFs [11,12,15,17]. For example, in the case of halofantrine [12] and fenofibrate [15], dose loading increased to 150% compared to solubility at 37 °C, whereas for simvastatin [11] and R4040 [17], dose loading was increased to 200% compared to solubility at 37 °C. This indicates that many model drugs already show a reasonably high solubility in the lipid vehicles or a lower therapeutic dose compared to venetoclax. In fact, the choice of lipid vehicle is important, as indicated by the better ability to obtain and maintain higher supersaturated venetoclax concentrations in monoglycerides when compared to triglycerides. While venetoclax displayed an ability to supersaturate in all lipid vehicles, the final choice of lipid vehicle will be influenced by factors such as the overall required dose loading and the stability of the formulation over time. In our study, appropriate short-term stability was observed for the Peceol^®^ sLBF, where the concentration was maintained above the target of 50 mg/mL over a 2 week observational period with no macroscopic signs of instability. 

In the in vivo study, the highest absolute oral bioavailability was observed for sLBF, with 17.4% ± 5.5%. sLBF showed a 2.1-fold increased bioavailability relative to the Peceol^®^ suspension and a 3.8-fold increase when compared to the venetoclax powder capsule, which in the latter case was statistically significantly different (*p* < 0.05). Additionally, sLBF showed a faster absorption and shorter residence time in pigs. The results from the in vivo study were in line with observations from the in vitro lipolysis of sLBF, which suggested the combined effect of maintaining the drug within the absorbable lipid phase and the increased venetoclax concentration observed in the aqueous digestive phase. In a previous study with fenofibrate in minipigs, a sLBF with 150% drug load (compared to equilibrium solubility) was compared to a LBF suspension with the same drug load (100% dissolved and 50% suspended) [15]. The bioavailability and peak plasma concentrations of both sLBF and LBF suspension was similar [15]. In a separate study, the drug R3040 was studied as a sLBF with 200% drug load and a LBF suspension with the same drug load (100% dissolved and 100% suspended) in rats [17]. A similar bioavailability was observed for both formulations, while the peak plasma concentration tended to be higher in the case of the sLBF [17]. The results of the present study with the more atypical molecular characteristics and the higher degree of supersaturation of venetoclax, therefore, extended the range of application of the supersaturated formulation approach in lipid vehicles. 

The in vitro lipolysis showed a higher venetoclax concentration in the aqueous phase for the sLBF compared to a Peceol^®^ suspension and aqueous suspension after 60 min of digestion. After 60 min, venetoclax concentrations of 20.4 ± 1.2 µg/mL in fasted state-simulating digestion buffer were measured. This was in the range of the previously reported amorphous solubility of venetoclax under simulated intestinal fluid conditions in the fasted state of approximately 21–34 µg/mL depending on the pH [20]. Therefore, venetoclax concentrations near the maximum reported aqueous concentrations were reached with the developed sLBF. Such high venetoclax concentrations in this study agree with previous reports, which have shown that venetoclax can achieve high supersaturation in aqueous media [20]. In addition, venetoclax is a class III glass former (Figure 2, Figure S2, Table 1) with a high glass-forming ability, suggesting a reasonable stable supersaturated concentration [32]. Interestingly, in our study, a distinctly yellow ‘fourth’ phase, presumed to be a venetoclax-rich phase, was evident after ultracentrifugation at the bottom of the centrifugation tube (Figure 5). Given that previous studies have reported that concentrations above the amorphous solubility lead to a glass–liquid phase separation (GLPS) [20], this fourth phase after centrifugation may potentially represent a venetoclax-rich phase that has formed from high drug concentrations achieved during digestion in the aqueous phase. While this drug-rich phase is observed under non-sink conditions after ultracentrifugation, the likely impact of such elevated concentrations in vivo is to drive the absorptive flux which is especially important for BCS class IV drugs such as venetoclax. 

In addition, the lowest amount of solid was recovered throughout dispersion and digestion for sLBF. In fact, despite the increased kinetic stress due to dispersion and digestion, most venetoclax was recovered in the lipid phase for sLBF, which maintained drug supersaturation throughout the 60 min. While in previous studies supersaturated drug concentrations in in vitro lipolysis media have been reported [11], such high concentrations of drug within the lipid phase during digestion of a sLBF have not been reported previously for a sLBF. The high venetoclax concentration in the lipid phase may be attributed to the relatively low digestibility of Peceol^®^ (23.5 ± 0.9%) in the in vitro lipolysis experiment. For highly lipophilic drugs such as venetoclax, the presence of an undigested/partially dispersed lipid reservoir in vitro is likely to lead to an overall lower drug concentration within the aqueous digest phase. However, in the in vivo situation, such a lipid phase may be critical to maintain bio-enhancing effects by sustaining the aqueous phase concentration as venetoclax partitions from the lipid into the aqueous phase. An adjustment of the in vitro model to species-specific parameters such as enzyme activity, gastrointestinal volumes [33] and the addition of a gastric step [34,35] and an absorptive sink [36,37] may have offered additional insights into reliably correlating in vitro formulation characteristics with in vivo performance. 

The developed supersaturation protocol in the present study was an efficient method to supersaturate lipid vehicles within 30–60 min including heating and cooling. Previously published supersaturation protocols required ultrasonification, a heating period of approximately 3–5 h at 60 °C as well as cooling overnight at 37 °C [11,12,15]. Therefore, previous reports to induce supersaturation in lipids involved processing between 13 and 15 h [11,12,15]. The newly proposed protocol employed in this study therefore represents a more streamlined approach to increase dose loading in LBFs, which can be readily applied in a pre-clinical drug development setting, especially for instable ad hoc prepared formulations.

While this study demonstrated a proof of concept for a novel sLBF approach to enhance in vivo exposure, the study was not without limitations. Firstly, it was not possible to complete a full 3 × 3 way cross-over in the in vivo study due to a failure in dosing one of the experimental units the sLBF formulation. However, at an individual level and overall, the higher bioavailability of the sLBF was significant. In addition, while the solubility in biorelevant media was consistent, we have also reported venetoclax-specific variability in the estimate of venetoclax solubility in lipid excipients. It was not readily apparent why venetoclax showed the variable results. Nonetheless, it is an important point to note, as in early stages of drug development, variability (especially between batches) is likely to be a major consideration given relatively limited experience with the drug. Finally, we acknowledge that the findings presented herein are more suited to a pre-clinical environment, where a focus on maximising exposure for safety assessment is a key priority. The approach outlined allows increasing dose loading in lipid excipients without any needed advanced processing which is normally involved for a technological approach that routinely requires a large amount of drug substance to produce formulations with an appropriate short-term stability. Clearly more work is required to explore long-term stability as well as the possible improvement of supersaturation stability considering the incorporation of stability and performance-enhancing excipients such as polymeric stabilisers (precipitation inhibitors) to support stabilisation of the supersaturated state in the formulation and upon dispersion and digestion.

## 5. Conclusions

The present study investigated the potential of a sLBF approach to increase the oral bioavailability of the poorly water- and lipid-soluble compound venetoclax. A rapid and efficient supersaturation protocol was developed and sLBFs were successfully created, leading to increased dose loadings of between 1.2-fold and 67.9-fold. A higher degree of supersaturation was observed in monoglycerides compared to triglycerides. While the supersaturated concentrations were high, a therapeutically relevant dose was only achieved in the long chain monoglyceride Peceol^®^. A Peceol^®^ sLBF was a viable approach for dosing beyond Rule-of-Five drugs in a pre-clinical setting, leading to an increased in vivo exposure of approximately 4-fold compared to a venetoclax powder capsule. Compared to the corresponding LBF suspension, an approximately 2-fold increase was demonstrated for sLBF. The in vitro lipolysis provided a mechanistic basis for explaining the sLBF performance relative to a control LBF and aqueous suspension in terms of higher solubility in the aqueous digest phase and the ability to maintain supersaturated drug concentrations in the lipid phase even after 60 min of digestion. Consequently, the present study demonstrated that a sLBF approach is a viable approach to increase the bioavailability of high-molecular-weight, hydrophobic and/or lipophilic beyond Rule-of-Five drugs. The findings of this study are of particular relevance to pre-clinical development, where an sLBF approach offers advantages relative to alternative bio-enabling approaches in terms of ease of preparation of high-dose-load formulations with the potential to maximise in vivo exposure.

## Figures and Tables

**Table 1:** Physicochemical properties and in vivo data of venetoclax.

Physicochemical Characteristics	
Log*P* ^1^	5.5
Molecular weight [g/mol] ^1^	868.44
BCS class ^2^	IV
Food effect ^1^	3.4-fold c_max_ and AUC increase with a light meal 5-fold c_max_ and AUC increase with a high fat meal
Melting point [°C]	138.61 ± 0.12 °C ^3^ 138 °C onset ^4^
Glass-forming ability ^3^	III
Venclyxto^®^ tablet core excipients ^5^	Copovidone Polysorbate 80 Colloidal anhydrous silica Anhydrous calcium hydrogen phosphate Sodium stearyl fumarate

^1^ Obtained from FDA Venclexta^®^ (venetoclax) clinical pharmacology and biopharmaceutics review [18]; ^2^ obtained from Emami Riedmaier et al. [20]; ^3^ experimentally determined by DSC for venetoclax batch 1 (Figure S1, Figure 2, Figure S2); ^4^ obtained from EMA, CHMP assessment report, Procedure No. EMEA/H/C/004106/000 [19]; ^5^ obtained from Section 6.1 of the summary of product characteristics of Venclyxto^®^ as of 09/2018.

**Table 2:** Biorelevant solubility of venetoclax at 37 °C (mean ± SD; n = 3).

Biorelevant Solubility [µg/mL]			
Media	Batch 1	Batch 2	Batch 3
FaSSIF	5.5 ± 1.0	5.2 ± 0.8	4.8 ± 0.6
FeSSIF	26.3 ± 1.8	30.7 ± 4.0	28.2 ± 0.7

**Table 3:** Apparent solubility of venetoclax in lipid excipients at 37 °C (mean ± SD; n = 3).

Solubility in Lipid Excipients [mg/mL]			
Excipient	Batch 1	Batch 2	Batch 3
Olive oil	2.5 ± 0.2	0.3 ± 0.1	0.4 ± 0.1
Peceol^®^	19.4 ± 2.0	2.9 ± 0.2	4.3 ± 0.5
Captex^®^ 1000	0.4 ± 0.1	0.7 ± 0.1	1.0 ± 0.2
Capmul MCM^®^	6.1 ± 0.4	3.7 ± 0.5	5.9 ± 0.4

**Table 4:** Thermal properties of venetoclax. Enthalpy of fusion (Δ*H*_fus_), melting point (*T*_m_) and entropy of fusion (Δ*S*_fus_).

Batch	Δ*H*_fus_ (kJ/mol)	*T*_m_ (°C)	Δ*S*_fus_ × 10^−2^ (kJ/mol/K)
1705150	19.9 ± 0.7	138.6 ± 0.1	4.8 ± 0.2
180620	18.4 ± 0.4	140.2 ± 0.1	4.5 ± 0.1
11777 main peak	9.0 ± 1.7	143.2 ± 0.2	2.2 ± 0.4
11777 minor peak	2.7 ± 1.1	129.1 ± 0.2	0.7 ± 0.3

**Table 5:** Fold difference between the supersaturated venetoclax concentrations reached at 70 °C and the measured solubility for olive oil, Peceol^®^, Captex^®^ 1000 and Capmul MCM^®^, where n = 1 for initial screening.

Fold Difference			
Excipient	Batch 1	Batch 2	Batch 3
Olive oil	1.3	18.6	12.5
Peceol^®^	3.5	67.9	12.1
Captex^®^ 1000	10.1	28.2	3.7
Capmul MCM^®^	22.3	10.6	5.9

**Table 6:** Pharmacokinetic parameters of oral administration of 100 mg/pig venetoclax in male landrace pigs. Venetoclax was administered as a powder capsule, a Peceol^®^ suspension, and a supersaturated Peceol^®^ solution (sLBF) (n = 3, except sLBF, where n = 2). *t*_max_ and MRT are given as median (range), all other parameters as the mean ± SD.

Pharmacokinetic Parameter	Powder Capsule	Peceol^®^ Suspension	sLBF
*c*_max_ [ng/mL]	162.67 ± 99.24	355.17 ± 205.40	1032.52 ± 165.03
*t*_max_ [h] (range)	12 (10–12)	8 (7–9)	5 (4–6)
AUC 0 h–inf. [μg* h/mL]	2.00 ± 0.89	3.59 ± 1.35	7.59 ± 2.40
MRT [h] (range)	14.46 (13.67–15.53)	11.90 (10.21–12.43)	8.77 (7.13–10.41)
MAT	10.58 (9.79–11.65)	8.02 (6.33–8.55)	4.89 (3.25–6.53)
F_rel_ [%] ^1^	100.00	233.24 ± 183.80	429.46 ± 151.06
F_abs_ [%]	4.56 ± 2.03	8.19 ± 3.08	17.35 ± 5.48

^1^ Relative to the powder capsule.

## Supplementary Materials

The following are available online at https://www.mdpi.com/1999-4923/12/6/564/s1, Figure S1: DSC of venetoclax batches. Given are the melting temperatures and enthalpy of fusion (mean ± SD; n = 3). Figure S2: Hot-stage microscopy of venetoclax batch 1 under cross-polarised light. Pictures were taken at various temperatures during heating from room temperature to 165 °C (10 °C/min) and during cooling to 25 °C (20 °C/min).
