# Supplementary material for: Supersaturated Lipid-Based Formulations to Enhance the Oral Bioavailability of Venetoclax
Source: Pharmaceutics. 2020 Jun 18;12(6):564. doi: 10.3390/pharmaceutics12060564 (PMC7355533; doi:10.3390/pharmaceutics12060564)
Supplement: Supplementary file 1 [file pharmaceutics-12-00564-s001.pdf]

pharmaceutics

# Supplementary Materials: Supersaturated lipid-based formulations to enhance oral the bioavailability of venetoclax

**Niklas J. Koehl, Laura J. Henze, Martin Kuentz, René Holm and Brendan T. Griffin**



| Supporting information | Page |
|---|---|
| Figure S1. DSC of venetoclax batches. | 1 |
| Figure S2. Hot-stage microscopy of venetoclax batch 1 during heating and cooling. | 2 |



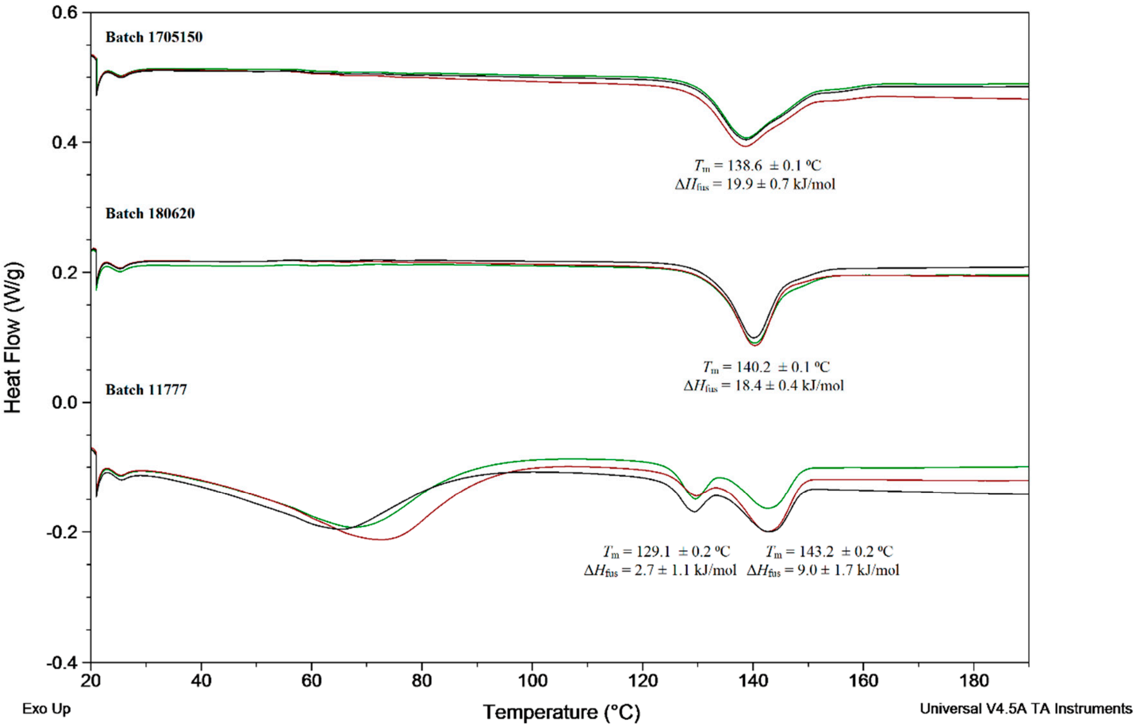


**Figure S1.** DSC of venetoclax batches. Given are the melting temperatures and enthalpy of fusion (mean ± SD, n = 3).

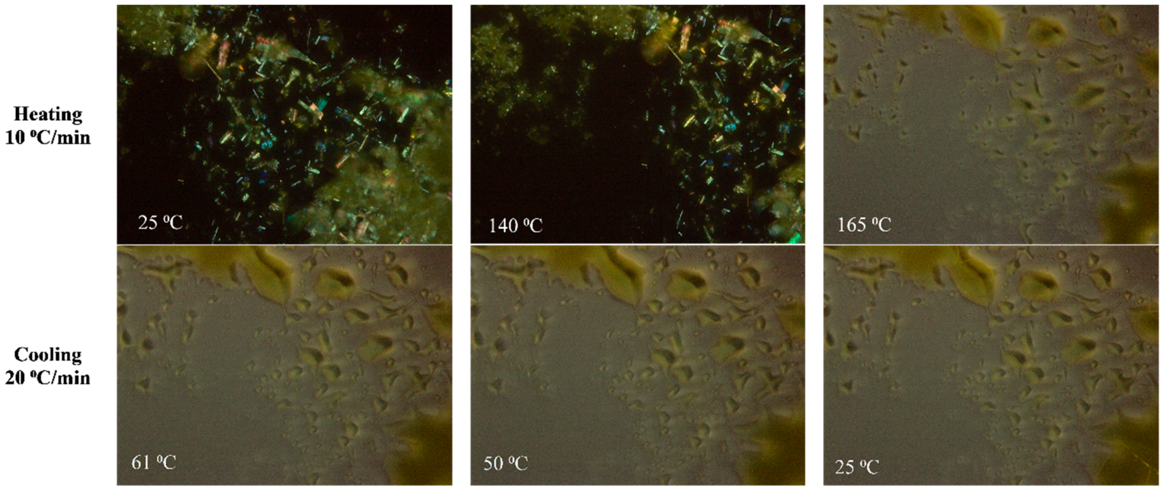

**Figure S2.** Hot-stage microscopy of venetoclax batch 1 under cross-polarised light. Pictures were taken at various temperatures during heating from room temperature to 165 $^0$C (10 $^0$C/min) and during cooling to 25 $^0$C (20 $^0$C/min).

© 2020 by the authors. Submitted for possible open access publication under the terms and conditions of the Creative Commons Attribution (CC BY) license (http://creativecommons.org/licenses/by/4.0/).
